# Revitalization in Traumatized Immature Teeth With External Inflammatory Root Resorption: A Systematic Review

**DOI:** 10.1111/edt.70082

**Published:** 2026-05-20

**Authors:** Ghina Al Khourdaji, Timothy Fasham, Samantha Elbhar, Kerstin M. Galler, Tchilalo Boukpessi

**Affiliations:** ^1^ Department of Restorative Dentistry Pitié Salpétrière Hospital (AP‐HP) Paris France; ^2^ Department of Prosthodontics Charles‐Foix Hospital (AP‐HP) Ivry‐sur‐Seine France; ^3^ UMR Santé Orale – UMR 1333 University Paris Cité Montrouge France; ^4^ Department of Operative Dentistry and Periodontology University Hospital Erlangen, University of Erlangen‐Nuremberg Erlangen Germany; ^5^ Department of Operative Dentistry and Endodontics Université Catholique de Louvain, Cliniques Universitaires Saint luc Bruxelles Belgium

**Keywords:** dental trauma, external inflammatory root resorption, pulp revitalization, regenerative endodontic procedures

## Abstract

**Introduction:**

This study aimed to evaluate whether revitalization can be indicated in the treatment of traumatized immature teeth with external inflammatory root resorption (EIRR).

**Methods:**

The protocol of the review was developed in accordance with the PRISMA checklist and registered in the PROSPERO database (CRD42023446609). Four electronic databases (PubMed, EMBASE, Scopus, Web of Science) were used for literature search. All studies that discussed the use of revitalization in the treatment of traumatized immature teeth with EIRR were included, and the quality of the assessment was evaluated through Joanna Briggs Institute JBI Critical Appraisal Tools to assess the risk of bias.

**Results:**

Thirteen studies fulfilled the desired inclusion criteria. In total, 20 teeth in 17 patients were analyzed. The survival rate was 100%, and 85% of the teeth were classified as success. 85% of the teeth treated showed resolution of clinical signs and symptoms as well as periapical radiolucencies and, furthermore, an arrest of EIRR during the follow‐up period. One tooth had arrested EIRR followed by replacement root resorption. Increase in root length and width was not predictable and was found in 45% of the immature teeth.

**Conclusions:**

Revitalization might be a promising alternative treatment producing biological repair and improving prognosis in cases of EIRR in post‐traumatic immature teeth once it is confirmed that the resorptive process is arrested after disinfection measures. However, the main limitation of this systematic review was that nine publications were case reports that usually tend to report successful outcomes.

## 1 Introduction

As stated in the respective position statement of the European Society of Endodontology, root resorption involves the breakdown of dental hard tissue resulting from the action of odontoclasts [1]. Resorptions can either originate from the internal root canal wall or from the external root surface [1]. Among the external root resorptions, external inflammatory root resorption (EIRR) is often linked with severe forms of trauma such as intrusive luxation and avulsion followed by inadequate storage [1]. Souza et al. have reported in a meta‐analysis that in the event of replantation of avulsed teeth, EIRR is the second most common type of root resorption (23.2%) after external replacement root resorption (ERRR) (51%) [2].

Both EIRR and ERRR are a consequence of severe damage of root precementum and cells of the desmodontium due to traumatic impact. The key difference between these two types of external resorptions lies in the state of infection. EIRR develops after trauma‐induced pulp necrosis and subsequent bacterial colonization of the root canal. Treatment in EIRR‐affected teeth prevents the diffusion of bacterial toxins through the dentinal tubules to the peri‐radicular tissue due to the lack of a functional barrier by root cementum, which would otherwise activate clastic cells [1, 3, 4]. These cells can target the exposed dentin, starting the resorptive process: an exposure consequent to the damage of the precementum following severe trauma. ERRR, however, is merely induced by the traumatic damage, where clastic cells that are active during physiological bone remodeling start to resorb root dentin, again due to the destruction of the protective barrier [1, 2, 3, 5]. This implies that EIRR can be prevented or treated by thorough disinfection of the pulp space and root canal to eliminate infection [1], whereas no therapeutic intervention exists to influence ERRR [6].

Different treatment options have been suggested in the treatment of immature permanent teeth with external inflammatory root resorption. A long‐term apexification with calcium hydroxide followed by root obturation with gutta‐percha has been first suggested [7, 8]. A more recent alternative is a 4 mm thick apical plug of mineral trioxide aggregate (MTA) [9] again followed by obturation of the remaining canal space with gutta‐percha [3, 10]. Both options have been debated since they may compromise long‐term integrity of the tooth. On one hand, long‐term exposure to calcium hydroxide could interfere with the collagenous structure of the dentin [11], and on the other hand, neither apexification nor an apical plug allows for hard tissue apposition to achieve increased dentinal wall thickness [12].

In the past 12 years, several studies have been using revitalization, alternatively termed regenerative endodontic procedures (REP), which is intended to address the limitations of both previously described options [13, 14, 15]. This approach aspires to (re)generate a vital tissue inside the root canal [16], and was firstly aimed to treat pulp necrosis in immature permanent teeth with or without signs of apical periodontitis [16]. The expected outcome includes the resolution of clinical signs and symptoms, periapical healing, as well as induction of an increase in root length and thickness [12, 16], thus strengthening the compromised tooth. There are two sets of guidelines with common points that give advice on an adequate protocol: one from the European Society of Endodontology (ESE) [16], and another from the American Association of Endodontists (AAE) [17]. Furthermore, revitalization is recommended as a potential treatment in immature teeth with pulp necrosis in the S3 guideline on the treatment of pulpitis and apical periodontitis [18].

The aim of this systematic review is to assess whether revitalization is a suitable treatment for traumatized immature teeth with external inflammatory root resorption.

## 2 Material and Methods

The systematic review was conducted based on the guidelines proposed by the Preferred Reporting Items for Systematic Reviews and Meta‐Analyses (PRISMA) and was registered in the PROSPERO database (CRD42023446609).

### 2.1 Formulating the Review Question

The review question was constructed on the population, intervention, comparison, and outcome (PICO) framework: can revitalization be indicated in the treatment of traumatized immature teeth with external inflammatory root resorption?

### 2.2 Selection Criteria

Case reports, case series, case control studies, retrospective studies, and clinical studies published in all languages were considered. However, we limited our analysis to studies for which a complete and interpretable version was available—that is, in practice, articles in English. Studies reporting revitalization in immature and mature teeth were eligible, but only data relating to immature teeth were extracted.

The exclusion criteria were the following:

- Full text not available;
- Opinion articles;
- Letters to the editor;
- Literature reviews;
- Meta‐analyses;
- Animal studies;
- In vitro and ex vivo studies;
- Studies in which the type of tooth was not defined;
- Studies in which the data for immature teeth could not be separated from mature teeth;
- Studies using revitalization in the treatment of teeth affected by other types of root resorptions such as internal root resorption or cervical root resorption.

### 2.3 Literature Search

Literature was searched from inception up to December 2024, in four electronic databases: PubMed, Scopus, Web of Science, and Embase. The search was last run on February 12, 2025. Free‐text terms were combined with Boolean operators, and the PubMed search strategy also incorporated a relevant MeSH term. All the search details are reported in Table S1. In addition, a manual search of the references within the articles was performed to complete the database. Two reviewers (G.A.K., T.F.) selected independently the articles according to the inclusion and exclusion criteria. The relevant articles were selected by a first screening of the title, then of the abstracts and finally after reading the full texts. To resolve disagreements, a third reviewer (T.B.) was involved in the final decision depending on these criteria. The records were exported onto bibliographic software (EndNote X7 Basic‐Thomson Reuters, New York, USA) to compile the records and remove the duplicates.

### 2.4 Data Extraction

A data extraction form was created for this systematic review and included the following items: name and country of the first author, year published, type of study, demographic characteristics of the sample (age, sex), tooth number, stage of tooth maturation, type of trauma, extra‐alveolar time, storage medium, clinical examination, pulpal and periapical diagnosis, presence of a periapical lesion, type of resorption, time to resorption, intracanal medication, dressing time (weeks), type of scaffold, type of coronal plug, last check‐up (months), positive response to pulp sensibility tests, healing of periapical lesion, clinical outcome, radiographic outcome, and complications.

### 2.5 Risk of Bias

Two reviewers (G.A.K., T).F. assessed the risk of bias of the included studies using the Joanna Briggs Institute (JBI) Appraisal Checklists, selecting the appropriate tool respective to the study design (i.e., case series or case reports) [19, 20]. In cases of uncertainty or lack of agreement, a third reviewer (T.B.) was involved for decision making. For each study, checklist items were judged as “yes,” “no,” “unclear,” or “not applicable.” Studies were ranked as “low risk of bias” when 70% or more of answers corresponded to “yes,” “moderate risk of bias” when between 50% and 69% of answers corresponded to “yes,” and “high risk of bias” if 49% or less of answers corresponded to “yes” [21, 22]. Figures evaluating the quality assessment of the studies were generated with Review Manager 5.3 (RevMan 5.3, The Nordic Cochrane Centre, Copenhagen, Denmark).

### 2.6 Outcome Variables and Statistical Analysis

Due to the heterogeneity among the different study designs, it was not possible to perform a meta‐analysis.

## 3 Results

### 3.1 Literature Search

The PRISMA flow diagram (Figure 1) summarizes the literature search process. From searches in the four databases and across manual search, 749 articles were identified. The removal of duplicates resulted in 528 articles. Screening the titles and abstracts narrowed down the selected articles to 19. After applying the inclusion and exclusion criteria, 13 articles were included in this systematic review to assess if revitalization can be indicated in the treatment of traumatized immature teeth with external inflammatory root resorption. Within the 13 included articles, nine of them were case reports and four were case series. All studies were published in English. The selected studies either came from Israel [23, 24, 25, 26], Libya [27], China [28], Brazil [29], Greece [30, 31], France [3], USA [32], and Iran [33, 34].

**FIGURE 1:**
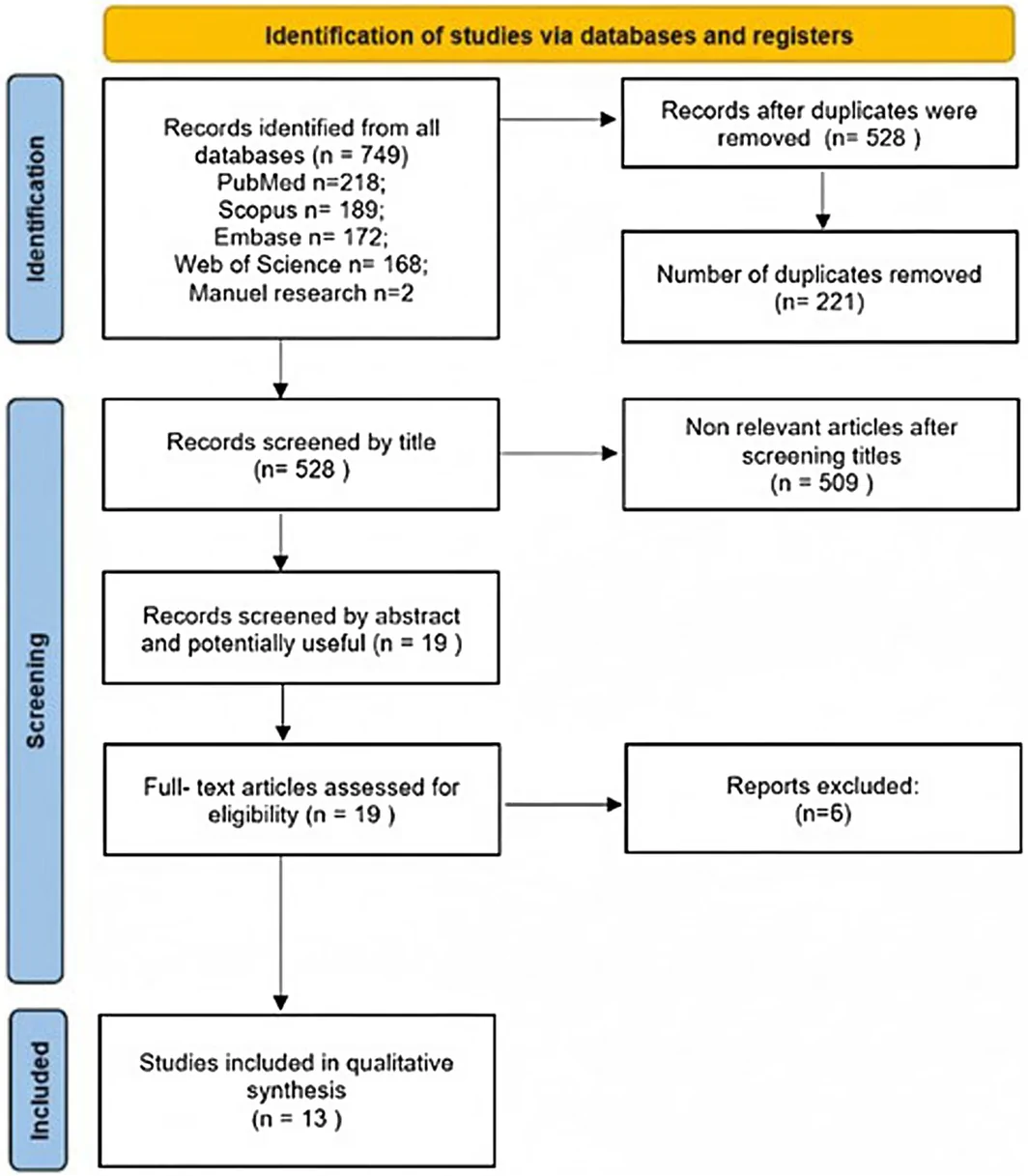
PRISMA flow diagram of the study.

The extraction data table for all the selected studies can be found in Table S2.

### 3.2 Data Extraction on Patient Demographic and Preoperative Clinical Characteristics

Table 1 sums up the patients' demographics and the clinical characteristics of the retained teeth within this study. In total, 20 teeth were retained in 17 patients: 11 teeth were compiled through case reports of 9 patients, and 9 teeth through case series of 8 patients. Regarding the sex of the patients, 45% of the teeth were from male patients, while 55% were from female subjects. The mean age across all patients was 10.1 years old with an age range of 7–24 years old.

**TABLE 1:** Patients' demographics and preoperative clinical characteristics of the retained teeth.

	Variables	Number of cases (teeth)	%
Mean age (years): 10.1	—	—	—
Sex	Female	11	55
	Male	9	45
Tooth type	Central incisor	19	95
	Lateral incisor	1	5
Tooth location	Maxillary	19	95
	Mandibular	1	5
Trauma type	Avulsion	9	45
	Extrusion	1	5
	Intrusion	2	10
	Subluxation	1	5
	Concussion	1	5
	UCF	7	35
	CCF	1	5
	Horizontal root fracture	1	5
	Not mentioned	1	5
Signs and symptoms	Absent	7	35
	Present	13	65
Periapical lesions	Absent	0	0
	Present	20	100

Of the 20 teeth with EIRR, 95% of the cases (19 teeth) were maxillary incisors, while one tooth was a mandibular incisor. A total of 17 teeth were diagnosed with pulp necrosis, one tooth with partial pulp necrosis, and two teeth with irreversible pulpitis. All recorded teeth had a periapical lesion and external inflammatory root resorption. Before treatment, two teeth exhibited a metallic sound following percussion testing, and two teeth had dyschromia.

Regarding the trauma types found in this systematic review, avulsion was the most frequent type (45%), followed by uncomplicated crown fracture (UCF) (35%).

When the extra‐alveolar period was reported, the mean period was 135 min. Regarding the storage medium, two teeth were stored in a dry environment, two teeth in milk medium, and two teeth in a combination of dry storage for a few minutes and milk medium.

### 3.3 Data Extraction on the Protocol of Revitalization

In only one case, treatment was completed in a single visit after using EndoVac (Sybron Endo) during irrigation, whereas in all remaining cases, two or more visits were needed to perform the treatment as intracanal medication was used. Calcium hydroxide was the most used intracanal medication and was selected for 12 teeth. Regarding the antibacterial paste that dressed the root canals, six cases used triple‐antibiotic paste (TAP: minocycline, metronidazole, ciprofloxacin), two used Trimix (metronidazole, cefuroxime axetil, ciprofloxacin), and three used double‐antibiotic paste (DAP: metronidazole, ciprofloxacin). The sequential use of calcium hydroxide and an antibiotic paste was described in four cases. The average dressing time was around three and a half weeks (3.7).

Sodium hypochlorite, with a concentration ranging from 1% to 6%, was commonly used except for one study which used 2% chlorhexidine as an irrigation solution. During the second visit, the use of a 17% EDTA solution during irrigation in 16 teeth was described.

A blood clot was induced in 13 cases, whereas 7 cases used platelet‐rich fibrin (PRF). Calcium silicate‐based materials from different manufacturers were selected for the coronal plug across this systematic review: 13 cases reported the use of mineral trioxide aggregate (MTA), 6 cases used Biodentine (Septodont), and 1 case used bioceramic root repair material (EndoSequence BC RRM, Brasseler).

### 3.4 Outcomes of Revitalization

The clinical and radiographical outcomes of 20 teeth treated with revitalization included in this study can be found in Table 2. The average follow‐up period for all the selected studies is approximately 22 months and a half (i.e., 1 year and 10 months and a half). In this study, all 20 teeth survived amongst which 17 teeth had a successful outcome (no signs and symptoms of inflammation, absence of periapical radiolucency) (85%). Three teeth were ranked as failures due to a reinfection with a new periapical radiolucency after 3 or 27 months.

**TABLE 2:** Clinical and radiographical outcomes of the 20 teeth treated with revitalization.

	Variables	Number of cases (teeth)	%
Treatment outcome	Surviving teeth	20	100
	Successful teeth	17	85
	Failed teeth	3	15
Pulp sensibility testing	Positive response	2	10
	Negative response	9	45
	Not mentioned	9	45
Postoperative radiographical outcome	Absence of periapical lesion after initial revitalization	17	85
	Intact periodontal ligament surrounding the root	13	65
	Replacement root resorption	1	5
	Calcification formed in the canal	6	30
	Continued root development	9	45

For 13 teeth, the periodontal ligament appeared intact around the root. Furthermore, one tooth which presented an arrested progression of EIRR exhibited ERRR, and the percussion test on this tooth produced a metallic sound indicative of ankylosis.

Six teeth (30%) in the study appeared to have calcified tissue formed within the root canal space and nine immature teeth had a continuous development of the root. Amongst the complications noted at follow‐up appointments, seven teeth featured the presence of a discoloration.

Furthermore, only two teeth reacted positively to pulp sensibility testing (i.e., thermal and/or electric testing), whereas nine teeth did not respond to these tests, the respective data were lacking for nine teeth.

### 3.5 Risk of Bias in the Study

Figures 2 and 3 illustrate the risk of bias within the selected case reports. Figures 4 and 5 display the risk of bias in the case series. The white cells in the latter indicate that these criteria were identified as not applicable. The risk of bias was ranked low for one case series and six case reports, moderate for three case series and two case reports, and high for one case report.

**FIGURE 2:**
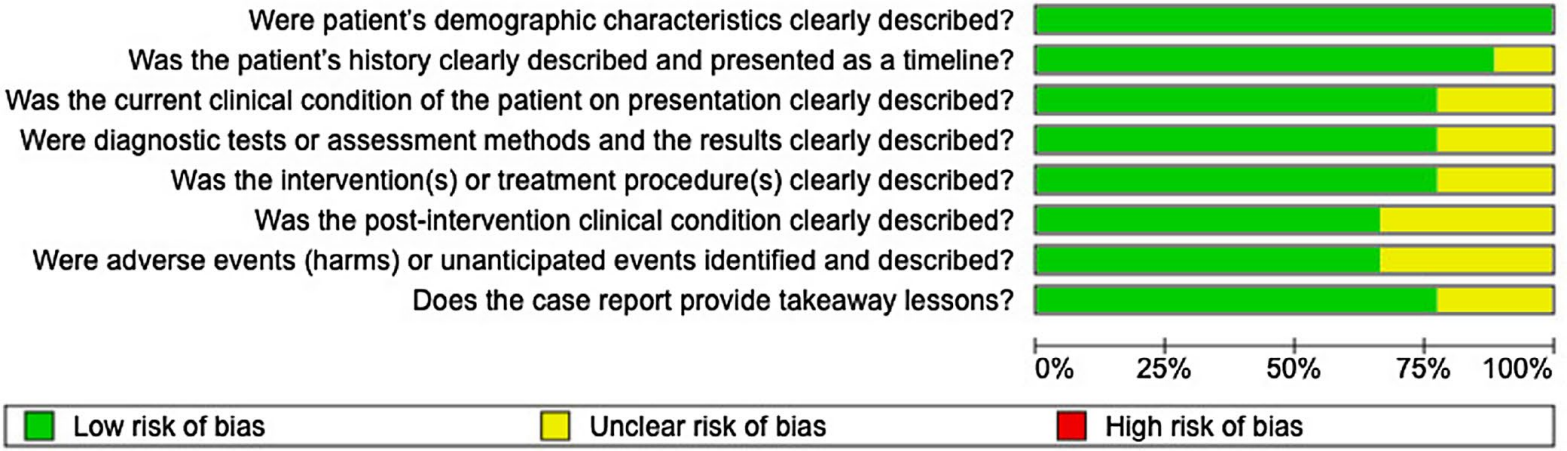
Case reports—risk of bias graph.

**FIGURE 3:**
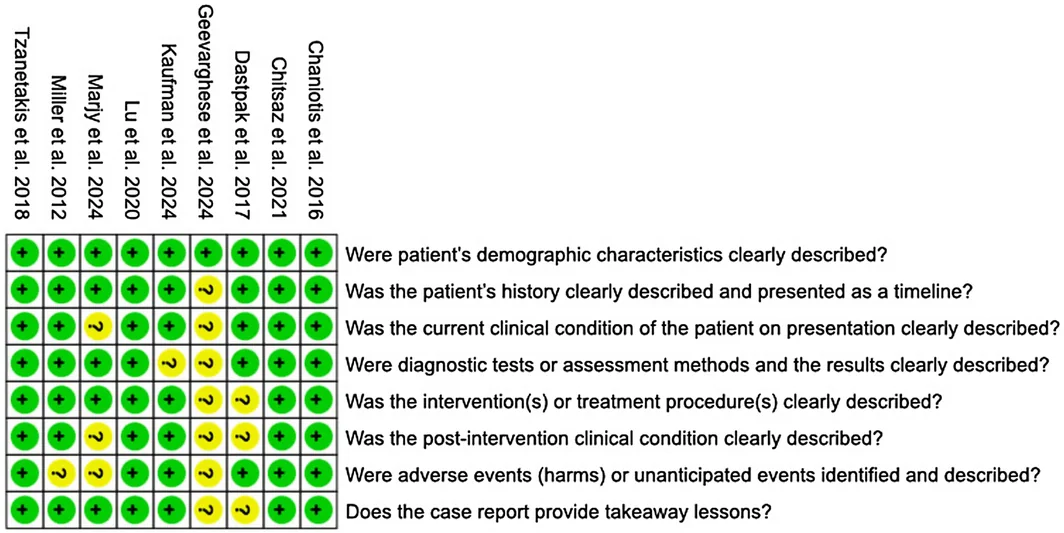
Case reports—risk of bias summary.

**FIGURE 4:**
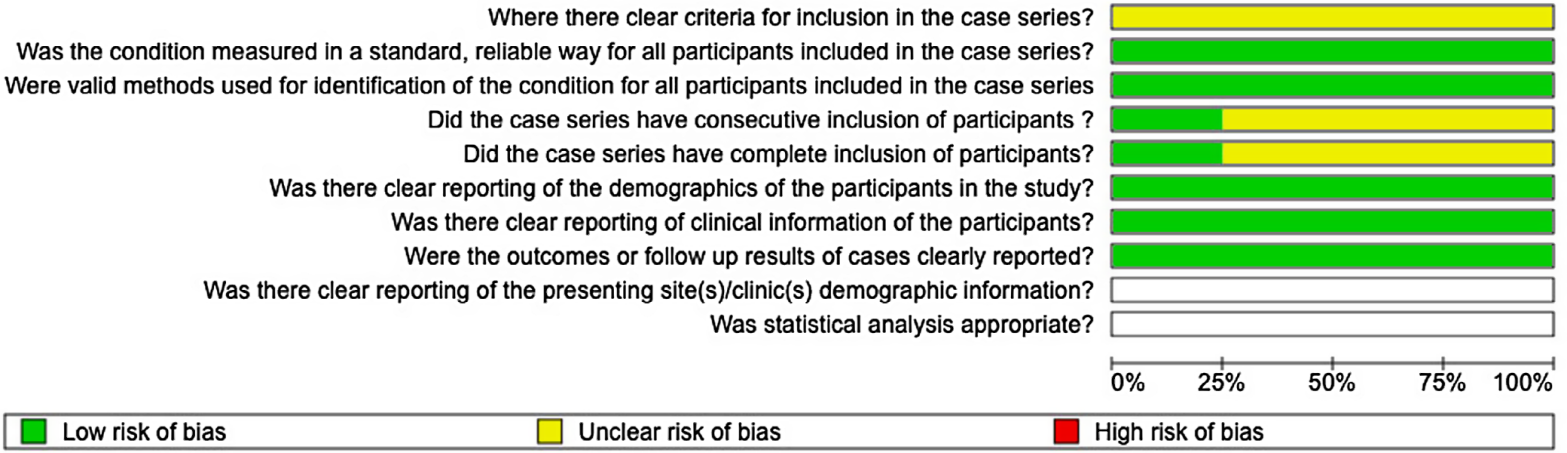
Case series—risk of bias graph.

**FIGURE 5:**
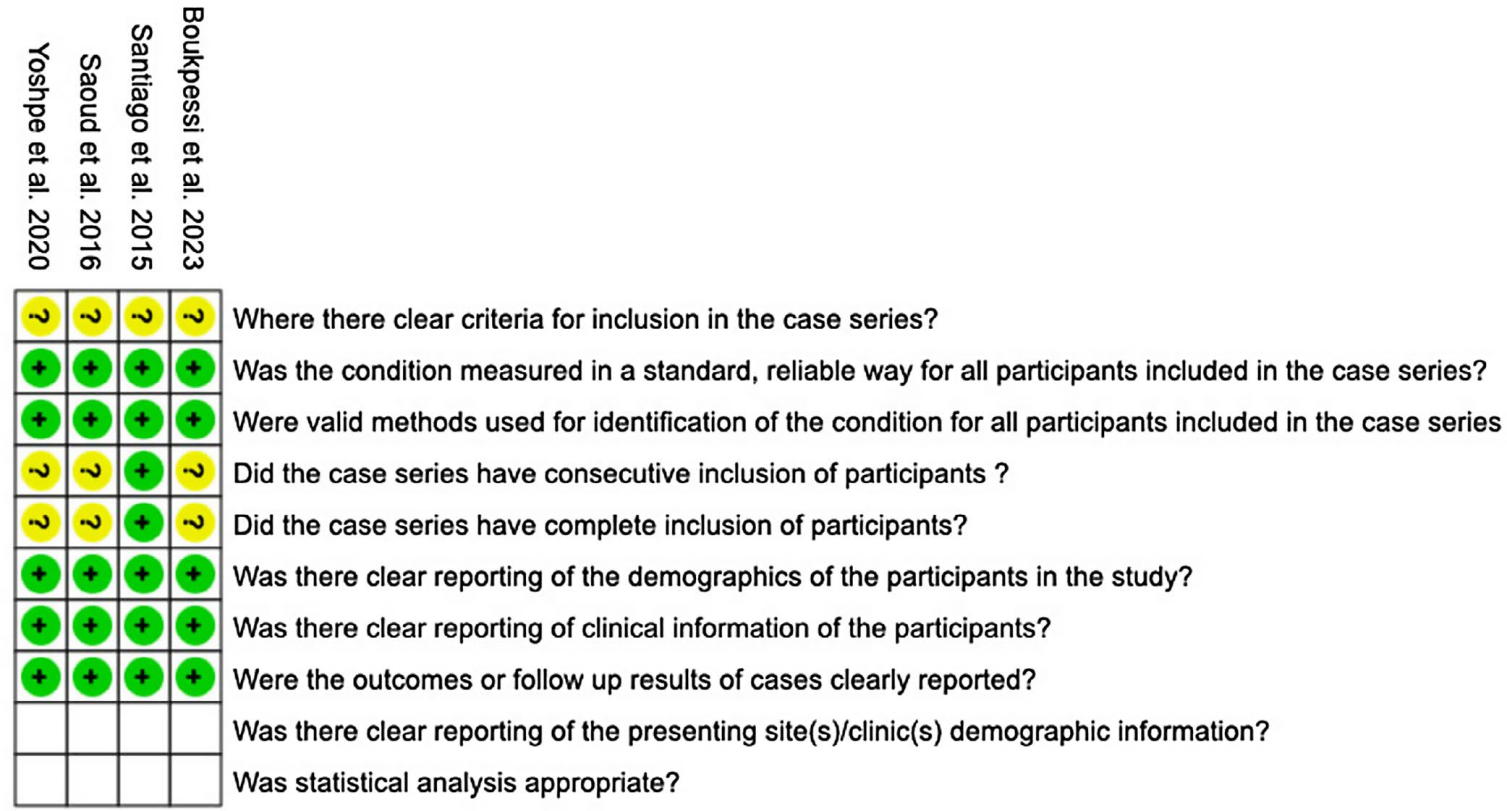
Case series—risk of bias summary.

## 4 Discussion

Up to now, no previous systematic studies have investigated the use of revitalization or regenerative endodontic procedures in the treatment of traumatized immature permanent teeth that exhibited external inflammatory root resorption. A recent systematic study has evaluated the use of revitalization but regardless of the tooth formation stage (i.e., immature or mature) and regardless of the type of root resorption (i.e., including both internal inflammatory and external inflammatory root resorption) [35]. However, due to the heterogeneity of the data, the pooled success rates could not be estimated by meta‐analysis.

Throughout this systematic study which included nine case reports and four case series, 20 teeth were treated with revitalization according to protocols based on the European Society of Endodontology (ESE) [16] or the American Association of Endodontists (AAE) [17] recommendations.

### 4.1 Issues Met With Immature Permanent Teeth

Pulp necrosis following a trauma will disrupt further development of the root. Depending on the stage of root development, the tooth will consequently be more prone to root fracture [8], due to thin canal walls, wide open‐apex [36], and an insufficient crown/root ratio [37]. For these reasons, performing conventional root canal therapy on fragile immature teeth is inadequate. Furthermore, the presence of root resorption adds to the difficulty of the endodontic treatment [38], and could affect the long‐term prognosis of the tooth [3].

Conventionally, the preferred treatment in cases of pulp infection in a immature permanent tooth with external inflammatory root resorption used to be a long‐term apexification with calcium hydroxide followed by root obturation with gutta‐percha when periodontal healing was observed [7, 8]. However, refreshing the dressing over an extended period requires the patient's compliance [8], and could also lead to a greater risk of root fracture due to the disruption of the collagenous structure and thus reduced mechanical strength of the dentin by calcium hydroxide [11].

More recently, a 4 mm thick apexification or apical plug of MTA [9], or bioceramic root repair material backfilled with gutta‐percha, has been suggested [10]. Although this approach has good success rates [37, 39, 40] and induces apical barrier formation [12, 41], it does not promote further root maturation [3, 12], or does so only to a limited extent [42]. Consequently, dentinal walls of the root canal remain thin. Therefore, the absence of tooth strengthening increases the risk of root fracture [12].

Conversely, revitalization has been recommended as an alternative for saving fragile immature permanent teeth. A retrospective study of Jeeruphan et al. reported that revitalization led to greater changes in root width and root length (28.2% and 14.9%) than MTA (0% and 6.1%) or calcium hydroxide (1.5% and 0.4%) apexification [42]. This difference in outcome could explain the higher survival rates of treated immature permanent teeth with revitalization compared to apexification [39, 42]. Systematic reviews and meta‐analyses comparing the outcomes between pulp revitalization and apexification have reported high success and survival rates for both approaches [37, 43, 44], without a significant difference suggesting in the overall outcome between them [43]. Nevertheless, an umbrella review highlighted that the certainty of evidence remains low for both outcomes due to the limitations of the included systematic reviews in that study [45].

Moreover, in the past few years, case reports and case studies have suggested the favorable use of revitalization to treat both traumatized immature [3, 23, 27, 28, 29, 30, 31, 32, 33, 34] and mature [23, 29] permanent teeth with EIRR. A recent clinical study [46] confirmed this trend that revitalization could contribute to arresting EIRR and enable positive quantitative changes in root dimension. Nonetheless, the recent endodontic S3‐level clinical practice guidelines advise that it is not suggested to perform revitalization on mature teeth due to the low quality of supporting evidence [18]. Hence, mature teeth with EIRR should consecutively be treated with conventional root canal treatment. For this reason, data on the cases involving revitalization of mature teeth with EIRR were excluded from analysis.

### 4.2 Clinical and Radiographical Outcomes and Complications

According to the ESE position statement, success criteria for revitalization include the absence of signs or symptoms of inflammation, periapical healing, the increase of root thickness and length, and positive response to sensibility testing [16]. In the presence of EIRR, another success criterion would be the arrested development of root resorption as well as the deposition of hard tissue [33] which has been qualified as wound healing [28].

In this study, all teeth survived and most teeth (85%) presented periapical healing and arrested development of EIRR after initial revitalization. These results are in accordance with the primary goal of revitalization being the resolution of apical periodontitis according to Jung et al. [47]. However, three teeth in the present study were assessed as failed either due to reinfection [3, 25, 29], and a previous clinical study suggested that failure may result also from a secondary EIRR [46].

In addition, replacement root resorption appeared relatively uncommon among the included cases of this systematic review as only one tooth in this study was reported to be affected by this complication. In contrast, Lu et al. documented a higher proportion, with five cases of ERRR in their sample of 20 teeth, four of which were associated with an avulsed tooth (80%) and one with intrusion [46]. This aligns more closely to the existing literature. ERRR can be expected in avulsed teeth with an extra‐oral dry time of more than 60 min [46], since a study of Andreasen et al. reported a significant association between the frequency of ankylosis and extended dry time [48]. Moreover, one study reported that ERRR occurred more frequently following an avulsion (87%) [49].

In Table 2, 30% of the teeth showed intracanal accumulation of calcified tissue. Nonetheless, this prevalence is lower than that reported in previous studies where rates of 61.2% [50] and 65% [46] have been documented following revitalization. This calcified tissue may be more bone‐ or cementum‐like, not necessarily pulp‐ or dentin‐like tissue [27, 51, 52]. That could be an issue due to on one hand the low mechanical properties of the newly formed intra‐canal tissue, and on the other hand this tissue could complicate future root canal therapy following a failure of revitalization [52]. Therefore, although the prevalence observed in this review is lower, caution remains warranted, as this ratio is dependent on the chosen inclusion and exclusion criteria.

Another reported complication in this study is tooth discoloration. This occurred in 35% of the retained teeth (7 teeth), amongst which 4 teeth had TAP antibiotic paste dressing. A possible explanation could be the presence of minocycline in TAP paste, which has been reported to lead to unfavorable discolorations [53, 54]. Nonetheless, these seven teeth also had a coronal plug that included a layer of MTA. The presence of bismuth oxide in MTA has also been linked to causing tooth discoloration [54, 55], which is why tricalcium silicates and Biodentine have been suggested as alternative materials.

### 4.3 Treatment Timing and Progression of EIRR

According to the recorded data in Table S2, treatment was initiated only after diagnosis of EIRR which occurred generally between 3 and 12 months post‐trauma [3, 23, 24, 26, 28, 29, 31, 33], but in some cases as late as 3 [23] or 16 years [34]. This delay correlated with the progression of EIRR, as after 3 months, EIRR was already observed at moderate to advanced stages with multiple lacunae and sometimes perforations. These findings support the concept that EIRR is an infectious‐driven and rapidly progressing process [54, 56].

In cases of avulsion, the International Association of Dental Traumatology (IADT) guidelines recommend waiting for spontaneous revascularization in immature teeth. If it does not occur, apexification, revitalization, or root canal treatment should then be initiated. This recommendation reflects the need to weigh the risk of EIRR progression against the benefit of allowing natural revascularization, hence preserving tooth structure. Indeed, early and moderate stage EIRR in the included avulsed teeth were already reported after respectively only 1 month [29] and 7 weeks [32].

Nonetheless, not all EIRR cases in this review were linked to avulsion injuries. Other trauma types included mainly uncomplicated crown fracture (7 cases), complicated crown fracture, root fracture, concussion, subluxation, extrusion, as well as one case of intrusion. According to the IADT guidelines, revitalization is not specifically recommended in all these scenarios, as strict follow‐up and pulp monitoring are emphasized [57]. However, in the event of intrusion and lateral luxation of immature teeth, revitalization is recommended if spontaneous revascularization is excluded [57]. In the present study, moderate to advanced stages of EIRR were diagnosed 4–6 months after fractured and luxated teeth [24, 26].

Interestingly, 17 teeth were diagnosed with necrotic pulp at the same time as EIRR. This underlines that insufficient or inconsistent post‐trauma follow‐up and the resulting delay in initiating the appropriate endodontic treatment may have contributed to the progression of EIRR.

### 4.4 Choice of Disinfection and Scaffolds on Revitalization Success

Root canal disinfection is essential to eliminate bacterial toxins, which, after penetration through the dentinal tubules to the damaged root surface, stimulate clastic cells [4]. This helps in halting the resorptive process. Moreover, the disinfection step is necessary for dissolving infected and necrotic tissue remnants within the root canal to avoid treatment failure [12]. A recent study has suggested that adequate disinfection should lead to the disruption and degradation of the biofilm matrix [58].

In this study, four case reports did not fully adhere to the standardized irrigation protocol recommended by the ESE [16], and AAE [17] regarding revitalization. One case report performed the treatment in a single visit using an apical negative‐pressure irrigation system [31], another used chlorhexidine instead of sodium hypochlorite [32], and two case reports did not mention the use of EDTA [29, 34]. These deviations could affect the treatment outcome, as it has been shown, for example, that chlorhexidine has limited effectiveness in dissolving biofilm [59, 60], cytotoxic effects on SCAP, as well as disrupting cell adhesion to dentin [54]. Although sodium hypochlorite is an effective proteolytic agent in disrupting biofilm [61], a high concentration of sodium hypochlorite (i.e., 5.25% instead of the 1.5%–3% advised in the guidelines) can disrupt cell adhesion [62] and reduce stem‐cell survival [63]. Nonetheless, these effects are diminished following 17% EDTA conditioning [62, 63]. Another advantage of EDTA is the release of growth factors which would promote the stem cells' adhesion, migration and differentiation [62], thereby favoring the formation of new tissue inside the root canal and thus treatment outcome. More precisely, according to Kaufman and Kahler these factors can diffuse either to the canal lumen or the root surface due to the damaged cementum [24]. Hence, the repair process can be expected as these bioactive molecules would help in the recruitment of stem cells within the periodontal ligament and bone [24].

Regarding the intra‐canal dressing, most teeth in the study were treated with calcium hydroxide, which is highly recommended over antibiotic pastes in the ESE guideline [16]. Conversely, the AAE suggests using both alternatives [17]. Aside from the discoloration risk, other disadvantages to antibiotic paste include toxicity to stem cells [64], a possibility of developing resistance to antibiotics or causing an allergic reaction [61], and attenuating the efficiency in the release of growth factors after EDTA conditioning [65].

During revitalization or regenerative endodontic procedures, different scaffold alternatives have been suggested in the guidelines: blood clot in the ESE statement [16], as well as PRP, PRF or AFM in the AAE [17]. In the extraction table (Table S2), blood clot was the most commonly used scaffold (13 teeth), followed by PRF (7 teeth). According to Wikström et al., the lack of bleeding is reported as the major cause of failure of revitalization (25.1%) [66]. That issue could be caused by the use of a vasoconstrictor (i.e., epinephrine) during the second visit, although there is a lack of evidence supporting that matter [16]. In this study, most cases clearly described the use of local anesthesia without vasoconstrictor (i.e., 19 teeth), and no cases have reported the lack of bleeding after irritation of the apical tissue when blood clot formation was intended. Furthermore, descriptions of apical closure in the retained teeth of this study are sparse. Nonetheless, Murray et al. has reported that PRF or PRP scaffolds lead to a more frequent apical closure (85%) than with a blood clot (58%), along with a higher prevalence of periapical healing (100%) than with a blood clot (89%) [67]. Conversely, another study has pointed out that there were no differences on the outcome (i.e., periapical healing and apical closure) between PRR, PRP and blood clot [68]. The same study also documented an increased prevalence of root canal calcification with blood clot compared to the use of other scaffolds [68]. Moreover, an umbrella review concluded that the certainty of evidence was insufficient to determine whether any alternative scaffold is consistently superior to blood clot [69].

## 5 Limitations

One of the limitations of the current study is the low number of included teeth (*n* = 20) and the predominance of case reports or case series. These are likely to publish cases where the treatments have been successful. Hence, the findings are primarily exploratory and descriptive and should be interpreted with caution, as this could lead to a biased point of view on the outcomes of a treatment by the lack of transparency. As a result, it is difficult to decipher which protocol parameters could really influence the success of revitalization. In addition, this review was limited to English‐language full‐text publications which may have introduced language bias, and grey literature was not searched. These restrictions may have resulted in the omission of relevant cases published in other languages, as well as unpublished studies.

Another limitation is that there is a lack of standardization within the studies regarding the procedures performed, the time gap between patient recalls, or the follow‐ups assessment (i.e., 2D or 3D radiographical exam). For instance, only four studies have evaluated the treatment outcome with a cone beam computed tomography (CBCT) [3, 24, 28, 33], whereas the other studies used periapical radiographs. Although CBCT provides detailed three‐dimensional insights into structures, which may be beneficial for follow‐ups of EIRR in the vestibulo‐lingual plane, the use of this exam in children should be limited according to the ESE statement [70]. A suggested approach, used by Lu et al. [46], to analyze changes in root dimensions in teeth with EIRR following revitalization involves assessing the radiographical root area (RRA) both at the pre‐operative and follow‐up appointments. This method requires the use of an image processing software to calculate the difference between total root area and the root canal space [71], and it has been reported that this technique is highly reproducible and avoids arbitrary measurements [71]. Hence, this approach appears promising to be implemented in future case reports of traumatized teeth with EIRR and treated with revitalization, especially since these teeth are predominantly single rooted.

Furthermore, another limit is that not all studies were internally ranked with a low risk of bias, which ultimately could compromise the reliability of these studies. All in all, this variability can influence the outcome and hinder the decision making since case reports or case series present a low level of evidence in Evidence‐based medicine (EBM). On one hand, to ensure high quality of data for further analysis, careful patient selection and treatment performed according to statements have been suggested [3]. On the other hand, in order to provide evidence, pragmatic controlled trials have been suggested as an approach to enable the practitioner to have freedom of choice on performing the treatment [72].

## 6 Conclusion

Despite the limitations of the systematic review, revitalization or regenerative endodontic procedures appear to show a promising outcome in the treatment of traumatized immature permanent teeth with external inflammatory root resorption once it is confirmed that the resorptive process is arrested after disinfection measures. In most cases, clinical and radiographical examinations have shown a resolution of clinical symptoms and signs, as well as periapical healing and arrest of EIRR. Additionally, nearly half of the reported immature teeth presented further root development. These findings should be interpreted with caution given the limited number of included teeth and the predominantly case‐based nature of the evidence. In the event of failure, root canal therapy or apexification can serve as a second‐line treatment option. Future well‐designed controlled trials or studies following statements are necessary to assess the long‐term complications and provide evidence that revitalization can be a viable alternative to apexification in managing EIRR.

## Author Contributions

**Ghina Al Khourdaji:** methodology, data curation, writing – original draft. **Timothy Fasham:** data curation, writing – review and editing. **Kerstin M. Galler:** writing – review and editing, supervision. **Samantha Elbhar:** methodology, data curation, supervision. **Tchilalo Boukpessi:** project administration, methodology, data curation, supervision, writing – review and editing.

## Funding

The authors have nothing to report.

## Conflicts of Interest

The authors declare no conflicts of interest.

## Supporting information


**Table S1:** List of combinations of MeSH terms and keywords for electronic database search strategy.
**Table S2:** Characteristics of the included studies.

## Data Availability

The data that support the findings of this study are available from the corresponding author upon reasonable request.
