# Supplementary material for: Revitalization in Traumatized Immature Teeth With External Inflammatory Root Resorption: A Systematic Review
Source: Dent Traumatol. 2026 May 20;42(6):840–51. doi: 10.1111/edt.70082 (PMC13743847; doi:10.1111/edt.70082)
Supplement: Supplementary file 1 [file EDT-42-840-s001.docx]

**Supplementary tables showing the list of combinations of MeSH terms and keywords for electronic database search strategy (S1) and the characteristics of included studies (S2)**

**Table S1:** List of combinations of MeSH terms and keywords for electronic database search strategy

| Database | Search strategy (December 2024) | N° |
| --- | --- | --- |
| PubMed | (("Tooth resorption" OR "resorption tooth" OR "root resorption" OR "resorption root" OR "dental root resorption " OR "inflammatory root resorption" OR "external inflammatory root resorption" OR "internal root resorption" OR "external root resorption" OR “resorption”) AND ("dental trauma" OR "dental traumas" OR "trauma dental" OR "tooth trauma" OR "trauma tooth" OR "dental injury" OR "dental injuries" OR "tooth injury" OR "injury tooth" OR "tooth injuries" OR "injuries tooth" OR "Dental pulp necrosis" OR "pulp necrosis" OR "necrosis" OR "necrotic" OR "nonvital" OR "non-vital" OR "Tooth, Nonvital" OR "apical periodontitis" OR " chronic apical periodontitis " OR "acute apical periodontitis " OR "Wounds and Injuries"[Mesh])) AND ("regenerative endodontics" OR "regenerative endodontic treatment" OR "regenerative endodontic procedures" OR "revascularization" OR "revitalization" OR "pulp revascularization" OR "pulp regeneration" OR "pulp revitalization" OR "regenerative endodontic" OR "pulp regenerative" OR “regenerative”) | 218 |
| Scopus | TITLE-ABS-KEY ( "Tooth resorption" OR "resorption tooth" OR "root resorption" OR "resorption root" OR "dental root resorption " OR "inflammatory root resorption" OR "external inflammatory root resorption" OR "internal root resorption" OR "external root resorption" OR “resorption” ) AND TITLE-ABS-KEY ( "dental trauma" OR "dental traumas" OR "trauma dental" OR "tooth trauma" OR "trauma tooth" OR "dental injury" OR "dental injuries" OR "tooth injury" OR "injury tooth" OR "tooth injuries" OR "injuries tooth" OR "Dental pulp necrosis" OR "pulp necrosis" OR "necrosis" OR "necrotic" OR "nonvital" OR "non-vital" OR "Tooth, Nonvital" OR "apical periodontitis" OR " chronic apical periodontitis" OR "acute apical periodontitis" ) AND TITLE-ABS-KEY ("regenerative endodontics" OR "regenerative endodontic treatment" OR "regenerative endodontic procedures" OR "revascularization" OR "revitalization" OR "pulp revascularization" OR "pulp regeneration" OR "pulp revitalization" OR "regenerative endodontic" OR "pulp regenerative" OR “regenerative” )) | 189 |
| Web of Science | "Tooth resorption" OR "resorption tooth" OR "root resorption" OR "resorption root" OR "dental root resorption" OR "inflammatory root resorption" OR "external inflammatory root resorption" OR "internal root resorption" OR "external root resorption" OR "resorption" ) AND TITLE-ABS-KEY ( "dental trauma" OR "dental traumas" OR "trauma dental" OR "tooth trauma" OR "trauma tooth" OR "dental injury" OR "dental injuries" OR "tooth injury" OR "injury tooth" OR "tooth injuries" OR "injuries tooth" OR "Dental pulp necrosis" OR "pulp necrosis" OR "necrosis" OR "necrotic" OR "nonvital" OR "non-vital" OR "Tooth, Nonvital" OR "apical periodontitis" OR "chronic apical periodontitis" OR "acute apical periodontitis" ) AND TITLE-ABS-KEY ( "regenerative endodontics" OR "regenerative endodontic treatment" OR "regenerative endodontic procedures" OR "revascularization" OR "revitalization" OR "pulp revascularization" OR "pulp regeneration" OR "pulp revitalization" OR "regenerative endodontic" OR "pulp regenerative" OR "regenerative" (All fields) | 168 |
| Embase | ("Tooth resorption" OR "resorption tooth" OR "root resorption" OR "resorption root" OR "dental root resorption" OR "inflammatory root resorption" OR "external inflammatory root resorption" OR "internal root resorption" OR "external root resorption" OR "resorption") AND ("dental trauma" OR "dental traumas" OR "trauma dental" OR "tooth trauma" OR "trauma tooth" OR "dental injury" OR "dental injuries" OR "tooth injury" OR "injury tooth" OR "tooth injuries" OR "injuries tooth" OR "Dental pulp necrosis" OR "pulp necrosis" OR "necrosis" OR "necrotic" OR "nonvital" OR "non-vital" OR "Tooth, Nonvital" OR "apical periodontitis" OR "chronic apical periodontitis" OR "acute apical periodontitis" OR "Wounds and Injuries") AND ("regenerative endodontics" OR "regenerative endodontic treatment" OR "regenerative endodontic procedures" OR "revascularization" OR "revitalization" OR "pulp revascularization" OR "pulp regeneration" OR "pulp revitalization" OR "regenerative endodontic" OR "pulp regenerative" OR "regenerative medicine") | 172 |

**Table S2:** Characteristics of the included studies

| Author  Country  Year | Study design | Gender | Age  (yrs) | Tooth  number | Type of trauma | Extra-alveolar period if avulsion (mins) | Storage  condition if avulsion | Clinical  examination | Pulpal and  periapical  diagnosis | Presence of  PAL | Type of  resorption | Time to  resorption |
| --- | --- | --- | --- | --- | --- | --- | --- | --- | --- | --- | --- | --- |
| Yosphe et al. Israel, USA 2020 | Case series | Case 1: M Case 2: F | Case 1: 11.5 Case 2: 9 | Case 1: #11 Case 2: #11 | Case 1,2: Avulsion | Case 1: NM Case 2: 60 | Case 1: NM Case 2: dry storage | Case 1, 2: Cold testing: - Percussion: tenderness & metallic sound Case 1: crown discoloration | Case 1, 2: PN & SAP | Yes | Case 1, 2: Advanced EIRR | Case 1: 3 yrs Case 2: 6 months |
| Saoud et al. Libya 2016 | Case series | Case 1: F Case 2: M | Case 1: 7 Case 2: 16 | Case 1: #21 Case 2: #11 | Case 1: Avulsion Case 2: UCF | Case 1: 40 | Case 1: 10 mins dry storage + 30 mins in milk | Case 1, 2: Cold testing: - Case 1, 2 Percussion: tenderness Case 2: Palpation: tenderness Fluctuant swelling | Case 1: PN & SAP Case 2: PN & AAA | Yes | Case 1: early stage EIRR Case 2: Perforating EIRR & IRR | Case 1: 6 weeks Case 2: 2 years |
| Lu et al. China 2020 | Case reports | F | 9 | #21 | Avulsion | 300 | Dry storage | Cold testing: - Palpation & percussion: tenderness Sinus tract Crown discoloration | PN & SAP | Yes | Perforating advanced EIRR | 4 months |
| Santiago, Brazil 2015 | Case series | Case 1: M Case 2: M | Case 1: 9 Case 2: 8 | Case 1: #11, #21 Case 2: #11 | Case 1: UCF for both teeth Case 2: Avulsion | Case 1: NA Case 2: 120 | Case 2: Milk storage | Case 1, 2: Cold testing: - Case 1,2: No tenderness to percussion & palpation | Case 1:  #11: PN & CAA #21: PN & AAP Case 2: PN & AAP | Yes | Case 1: #11 Advanced stage EIRR #21 early stage EIRR limited to apical third Case 2: Early stage EIRR | Case 1: 12 months Case 2: 1 month |
| Tzanetakis, Greece, 2018 | Case reports | M | 7 | #21 | Intrusion & UCF | NA | NA | Cold & electric testing: - Palpation & percussion: tenderness Swelling | PN & AAA | Yes | Moderate stage EIRR | 2 months |
| Chaniotis, Greece, 2016 | Case reports | F | 7 | #31 | Avulsion | 20 | Milk storage | Cold & electric testing: - Palpation & percussion: no tenderness | PN & AAP | Yes | Advanced EIRR | 6 months |
| Miller et al. USA, 2012 | Case reports | M | 9 | #21 | Avulsion | 270 | 3 mins dry storage & milk storage | Cold testing: + Palpation & percussion: no tenderness | AIP & AAP | Yes | Moderate stage EIRR middle third | 7 weeks |
| Chitsaz et al. Iran, 2021 | Case reports | F | 8 | #21 | CCF | NA | NA | Cold testing: + Percussion: tenderness | AIP + SAP | Yes | Advanced EIRR | 4 months |
| Dastpak, Iran, 2017 | Case reports | F | 24 | #21 | NM | NM | NM | Cold testing: - Palpation & percussion: tenderness Slightly discolored | PN + CAP | Yes | Advanced stage EIRR | 16 years |
| Boukpessi et al, France, 2023 | Case series | Case 1: F Case 2: M | Case 1: 7 Case 2: 7 | Case 1: #21 Case 2: #21 | Avulsion | NM | NM | Case 1, 2: Cold testing: - | Case 1: PN + SAP Case 2: PN + SAP | Yes | Case 1: Advanced stage EIRR Case 2: Moderate stage EIRR | Case 1: 3 months Case 2: NM |
| Kaufman et al, Israel, 2024 | Case report | F | 16 | #12, #21 | Concussion #12, Subluxation #21 | NA | NA | #12:  cold testing: + no percussion tenderness #21:  cold & electric testing: -  percussion tenderness | #12: Partial PN + AAP #21: PN + SAP | Yes | Moderate stage EIRR | 5 months |
| Marjy et al, Israel, 2024 | Case report | M | 9 | #11 | Extrusion, UCF, & Horizontal mid-root fracture | NA | NA | Cold testing: -  Percussion and palpation tenderness | PN + SAP | Yes | Early stage EIRR | 4 days |
| Geevarghese et al, Israel, 2024 | Case report | F | 9 | #11, #21 | Intrusion & UCF for both teeth | NA | NA | #11, #21:  Heat testing: -  percussion tenderness NM | #11, #21: PN + SAP | Yes | Moderate stage EIRR | 6 months |

| Author  Country  Year | Intracanal  medication | Dressing  time  (weeks) | Type of  scaffold | Type of coronal  plug | Last  follow-up  (months) | Positive  response  to sensibility tests | Clinical  outcome | Radiographic  outcome | Complications |
| --- | --- | --- | --- | --- | --- | --- | --- | --- | --- |
| Yosphe et al. Israel, USA 2020 | Case 1: Ca(OH)2/ Trimix Case 2: Trimix | Case 1: 4+3 Case 2: 3 | PRF | Biodentine | Case 1: 22 Case 2: 12 | Case 1, 2: No | Resolution of clinical signs  and symptoms Case 1: absence of metallic sound  Case 2: ankylotic sound on percussion | All survival cases (EIRR arrested, healing of PALs, 1 tooth with further root development and apical barrier, 1 tooth with ERRR) | Case 2: ERRR |
| Saoud et al. Libya 2016 | Case 1: TAP  Case 2:  Ca(OH)2/TAP | Case 1: 4  Case 2: 2 +2 | Blood clot | MTA | Case 1: 60 Case 2: 19 | Case 1, 2: No | Resolution of  clinical signs  and symptoms | EIRR arrested, healing of PALs, 1 with  further root development and thickening of walls, 1 with calcification and arrested root development after root fracture | Case 1: horizontal root fracture after further trauma |
| Lu et al. China 2020 | Ca(OH)2 | 2 | Blood clot | MTA | 30 | NM | Resolution of  clinical signs  and symptoms | EIRR arrested,  healing of PAL, root canal filled with hard tissue, thickening of dentinal walls | None |
| Santiago, Brazil 2015 | TAP | 4 | Blood clot | MTA | Case 1: 30 Case 2: 15 | NM | Resolution of  clinical signs  and symptoms except tooth #21 (case 1) | All survival cases (EIRR arrested, healing of PALs, all teeth had resorption area filled with hard tissue, 1 tooth with no change in root development, 1 tooth with further root development, 1 tooth reinfected and followed by RCT) | Case 1: #21 reinfection at 27 months follow-up months Case 1, 2: crown discoloration |
| Tzanetakis, Grece, 2018 | Ca(OH)2 | 2 | Blood clot | MTA | 30 | NM | Resolution of  clinical signs  and symptoms | EIRR arrested, healing of PAL, no further root development & some calcified deposits | None |
| Chaniotis, Grece, 2016 | Irrigation 6% NaOCl solution through EndoVac negative pressure & 17% EDTA | 1 visit | Blood clot | MTA | 24 | NM | Resolution of  clinical signs  and symptoms | EIRR arrested, healing of PAL, possible calcified tissue formed, further root development | Slight cervical discoloration |
| Miller et al. USA, 2012 | TAP | 6 | Blood clot | MTA | 18 | Yes | Resolution of  clinical signs  and symptoms | EIRR arrested, healing of PAL, further root development & apical closure | Cervical discoloration |
| Chitsaz et al. Iran, 2021 | DAP | 3 | PRF | MTA | 10 | NM | Resolution of  clinical signs  and symptoms | EIRR arrested, healing of PAL, limited evidence of further root development | Crown discoloration |
| Dastpak, Iran, 2017 | Ca(OH)2 | 3 | Blood clot | MTA | 12 | NM | Resolution of  clinical signs  and symptoms | EIRR arrested, limited evidence of further root development | Crown discoloration |
| Boukpessi et al, France, 2023 | Ca(OH)2 | 2 | Blood clot | Biodentine | 12 | Case 1: Yes Case 2: NM | Resolution of clinical signs and symptoms except case 2 | All cases survived with EIRR arrested, healing of PAL, further root development, apical closure/1 tooth with reinfection treated with apexification | Case 2: Reinfection at 3 months |
| Kaufman et al, Israel, 2024 | DAP/Ca(OH)2 | 3+3 | PRF | Biodentine | 36 | No | Resolution of clinical signs and symptoms | All teeth survived with EIRR arrested, thickening of dentinal walls, both with calcification in apical third | None |
| Marjy et al, Israel, 2024 | Ca(OH)2 | 2+6 | Blood clot | BC RRM | 3 | No | No initial resolution of clinical signs | Tooth reinfection treated with a second revitalization | Reinfection at 3 months |
| Geevarghese et al, Israel, 2024 | Ca(OH)2 | 2 | PRF | MTA | 20 |  | Resolution of clinical signs and symptoms | Both teeth survived with EIRR arrested, healing of PAL, further root development and apical closure | None |

*NM, not mentioned; NA, not applicable; EIRR, external inflammatory root resorption; IRR, internal root resorption; ERRR, replacement root resorption; UCF, uncomplicated crown fracture; CCF, complicated crown fracture; PAL, periapical lesion; PN, pulp necrosis; AIP, asymptomatic irreversible pulpitis; AAP, asymptomatic apical periodontitis; SAP, symptomatic apical periodontitis; AAA, acute apical abscess; CAP, chronic apical periodontitis; PRF, platelet-rich fibrin; MTA, mineral trioxide aggregate; BC RRM, bioceramic root repair material.*
